# Combining predominant polarity and affective spectrum concepts in bipolar disorder: towards a novel theoretical and clinical perspective

**DOI:** 10.1186/s40345-024-00336-9

**Published:** 2024-05-02

**Authors:** Francesco Bartoli, Gin S. Malhi, Giuseppe Carrà

**Affiliations:** 1 Department of Medicine and Surgery, University of Milano-Bicocca, Milan, Italy; 2 Academic Department of Psychiatry, Kolling Institute, Northern Clinical School, Faculty of Medicine and Health, The University of Sydney, Sydney, NSW Australia; 3 CADE Clinic and Mood-T, Royal North Shore Hospital, Northern Sydney Local Health District, Sydney, NSW Australia; 4 Department of Psychiatry, University of Oxford, Oxford, UK; 5 Oxford Uehiro Centre for Practical Ethics, Faculty of Philosophy, University of Oxford, Oxford, UK; 6 Division of Psychiatry, University College London, London, UK

## Abstract

This is an overview of recent advances on predominant polarity conceptualization in bipolar disorder (BD). Current evidence on its operationalized definitions, possible contextualization within the affective spectrum, along with its epidemiological impact, and treatment implications, are summarized. Predominant polarity identifies three subgroups of patients with BD according to their mood recurrencies: (i) those with depressive or (ii) manic predominance as well as (iii) patients without any preponderance (‘*nuclear*’ type). A predominant polarity can be identified in approximately half of patients, with similar rates for depressive and manic predominance. Different factors may influence the predominant polarity, including affective temperaments. More generally, affective disorders should be considered as existing on a spectrum ranging from depressive to manic features, also accounting for disorders with ‘*ultrapredominant*’ polarity, i.e., unipolar depression and mania. While mixed findings emerge on its utility in clinical practice, it is likely that the construct of predominant polarity, in place of conventional differentiation between BD-I and BD-II, may be useful to clarify the natural history of the disorder and select the most appropriate interventions. The conceptualization of predominant polarity seems to reconcile previous theoretical views of both BD and affective spectrum into a novel perspective. It may provide useful information to clinicians for the early identification of possible trajectories of BD and thus guide them when selecting interventions for maintenance treatment. However, further research is needed to clarify the specific role of predominant polarity as a key determinant of BD course, outcome, and treatment response.

## Introduction

Bipolar disorder (BD) is a severe and chronic condition, the burden of which is conferred largely by depression even though diagnostically it is characterized by mania. It affects at least 1% of the general population (McIntyre et al. 2020; Nierenberg et al. 2023; Vieta et al. 2018) and, unlike major depression (Malhi and Mann 2018), it involves males and females equally. While dimensional approaches to BD based essentially on the concept of a *spectrum* have been suggested by several proponents over the past four decades (Akiskal et al. 1977; Angst 2007; Klerman 1981; Malhi et al. 2018; Rosenthal 1975), current classificatory systems—in particular DSM-5 (American Psychiatric Association 2013, 2022) have maintained the somewhat controversial differentiation of depressive from bipolar and related disorders (Parker 2014), in lieu of the ‘*mood disorders*’ grouping, which has been gradually supplanted. Thus, according to the dominant psychiatric taxonomies, bipolar and depressive disorders are considered to be independent diagnostic entities, despite the near perfect overlap of the depressive phase in both clinical phenotypes and their respective diagnostic criteria. In other words, unipolar and bipolar depression are identical, and their commonality is further reinforced by the frequent diagnostic conversion of major depressive disorder to BD (Ratheesh et al. 2017), which also contributes to its considerable delay in diagnosis (Fritz et al. 2017). In addition, BDs are distinguished into type-I (BD-I) and type-II (BD-II), based on the presence or absence of manic episodes in the clinical history of patients (American Psychiatric Association 2013, 2022). Attempts have been made to show that several variables are differentially associated with BD-I as compared with BD-II disorders, such as gender, socioeconomic status, polarity of first and last episodes, occurrence of psychotic symptoms, age at onset, and symptom severity (Brancati et al. 2023; Serafini et al. 2019; Tondo et al. 2022a). However, this distinction has been questioned (Malhi and Bell 2022) and the matter is far from settled (Tondo et al. 2022b)—reflecting the challenge faced in clinical practice where the separation of the two putative kinds of depression is rarely achieved with sufficient confidence to be of prognostic value capturing again the phenomenological complexity of BD. For instance, the accuracy of BD-II diagnosis may be significantly reduced by both the problematic identification of lifetime history of hypomania and the difficult distinction of people with BD-II from those with a recurrent unipolar depression (Phillips and Kupfer 2013). Moreover, since the release of DSM-IV (American Psychiatric Association 1994), the same symptoms are listed for mania and hypomania, obfuscating any potential boundary between BD-I and BD-II (Benazzi 2007; Malhi and Berk 2014). In addition, the assumption that BD-II may represent a less severe phenotype than BD-I seems more related to the implicit characteristics of its definition (the absence of mania) than to the milder severity of its clinical course (Dell'Osso et al. 2015). In other words, severity is not a useful means of distinguishing subtypes.

Interestingly, although clinical conceptualizations of BD highlighted that people with this disorder are likely to spend more time with depression than with mania (Tondo et al. 2017), their clinical course may be rather heterogeneous and characterized by different individual propensities to depression and mania (Pallaskorpi et al. 2019; Sentissi et al. 2019). Consistent with this, it has been proposed that to attain a more accurate assessment of BD, it is prudent to consider as part of anamnesis whether the clinical history has been characterized by lifetime predominant depressive or manic/hypomanic episodes—a pattern that is self-evident when charted appropriately (Colom et al. 2015). Previous reviews highlighted the utility of predominant polarity as a clinical course specifier, facilitating its possible inclusion in current classification systems (Carvalho et al. 2014; García-Jiménez et al. 2019). Interestingly, preliminary studies have recently explored the neurobiological validity and plausibility of predominant polarity (Argyropoulos et al. 2023; Ruiz et al. 2022). However, its characterization within the affective spectrum may also complement previous theoretical views of BD (Angst 2007; Colom et al. 2015; Akiskal 1996; Ghaemi et al. 2022). In addition, it is possible that by differentiating BD according to an individual’s propensity towards depression or mania, it may be possible to derive novel cues for clinical practice, that may improve the personalized management of BD. The present paper is a selective and focused synthesis of recent advances on predominant polarity conceptualization, including its possible contextualization within the affective spectrum, epidemiological evidence, and treatment implications.

### Predominant polarity: definition and contextualization within the affective spectrum

The conceptualization of predominant polarity was introduced about 45 years ago by Angst (1978) who identified three subgroups of patients according to their mood recurrencies: patients with a clinical history of preponderantly manic or depressive episodes as well as those with a balanced proportion of both kinds of episodes, without any preponderance (the so-called ‘*nuclear*’ type). Thereafter, a more operationalized definition of predominant polarity in BD emerged from the ‘*Barcelona proposal*’ by Colom et al. (2006), following the ‘*2/3 rule*’. According to this, the ratio between lifetime depressive episodes and the overall number of mood episodes should be at least 2/3, for people with depressive predominance (DP). Conversely, a manic predominance (MP) can be claimed if at least 2/3 of lifetime mood episodes are manic/hypomanic. Finally, a nuclear BD (indeterminate polarity) is defined by the absence of either DP or MP. However, a less restrictive definition of predominant polarity was proposed later by Baldessarini et al. (Baldessarini et al. 2012). This is also known as the ‘*Harvard index*’ (Colom et al. 2015; Grover et al. 2021) and it is based on the estimate of the ratio between previous manic and depressive episodes, defining MP and DP if this ratio is higher or lower than one. Using this definition, the number of people with an indeterminate (nuclear) polarity would be reduced, those switching from one predominant polarity to another over the course of BD would be more (Colom et al. 2015). Considering that an indeterminate polarity has been associated with a specific clinical course, characterized by a greater number of relapses, mixed episodes, aggressive behaviors, and seasonality (Fico et al. 2022), the ‘*Barcelona proposal*’ is generally more used than the ‘*Harvard index*’. Nonetheless, current definitions did not account for mixed episodes, and their role in determining predominant polarity remains controversial. However, some researchers have proposed the creation of a fourth subtype, i.e., *the mixed phenotype* (Fico et al. 2023), which may also inform treatment if characterized sufficiently.

**Fig. 1:**
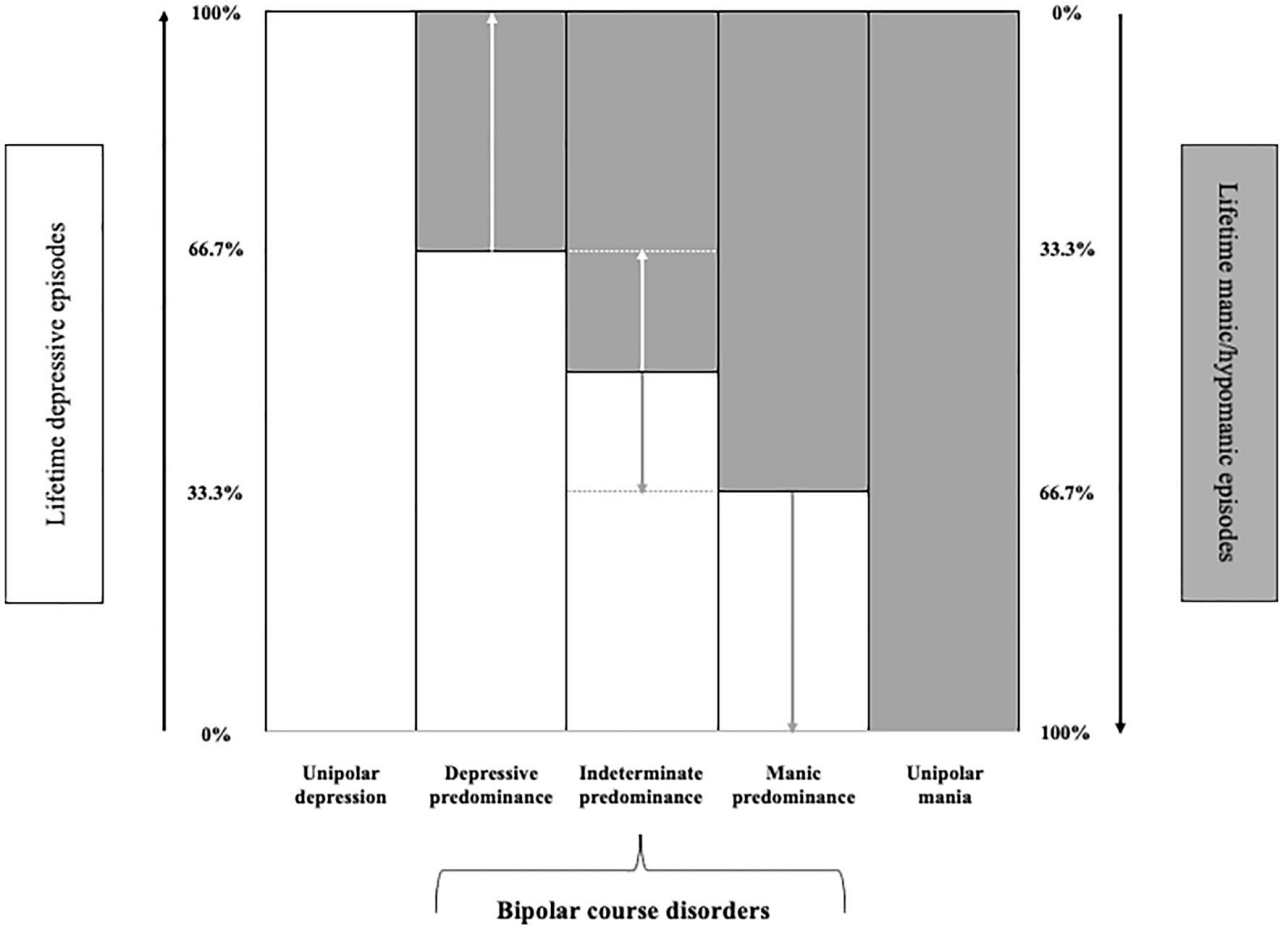
The affective spectrum -including both unipolar and bipolar course disorders- with different depressive/manic predominance (based on the Barcelona proposal). Rates of lifetime depressive episodes are shown in white and those of lifetime manic/hypomanic episodes in grey

Regardless of specific definitions, it is crucial to understand if the predominant polarity conceptualization may contribute to a more unitary and somehow simplified classification within the affective spectrum framework. The concept of *spectrum* is actually used to designate groups of heterogeneous clinical entities sharing some similarities (Alarcon et al. 1987). In the context of affective disorders, a spectrum-based classification model would represent the theoretical ground for a comprehensive psychopathological framework of different clinical phenotypes (Angst 2007; Angst and Cassano 2005; Ghaemi and Dalley 2014). Following this approach, affective disorders should be considered as existing on a continuum ranging from depressive to manic features. This includes disorders with a unipolar (*ultra-predominant*) course, at the opposite ends of the spectrum, namely unipolar depression (‘major depressive disorders’ according to DSM) and unipolar mania (whose epidemiological and nosological subsistence has been convincingly established) (Angst and Grobler 2015; Bartoli et al. 2023a; Bartoli 2023), as well as disorders with a bipolar course with manic, depressive, or indetermined predominance, according to the ‘*Barcelona proposal*’ (Fig. 1). All these phenotypes may embrace mood episodes either with or without psychotic features and should be further classified according to their severity, thus ultimately combining both categorical and dimensional approaches (Angst 2007).

**Fig. 2:**
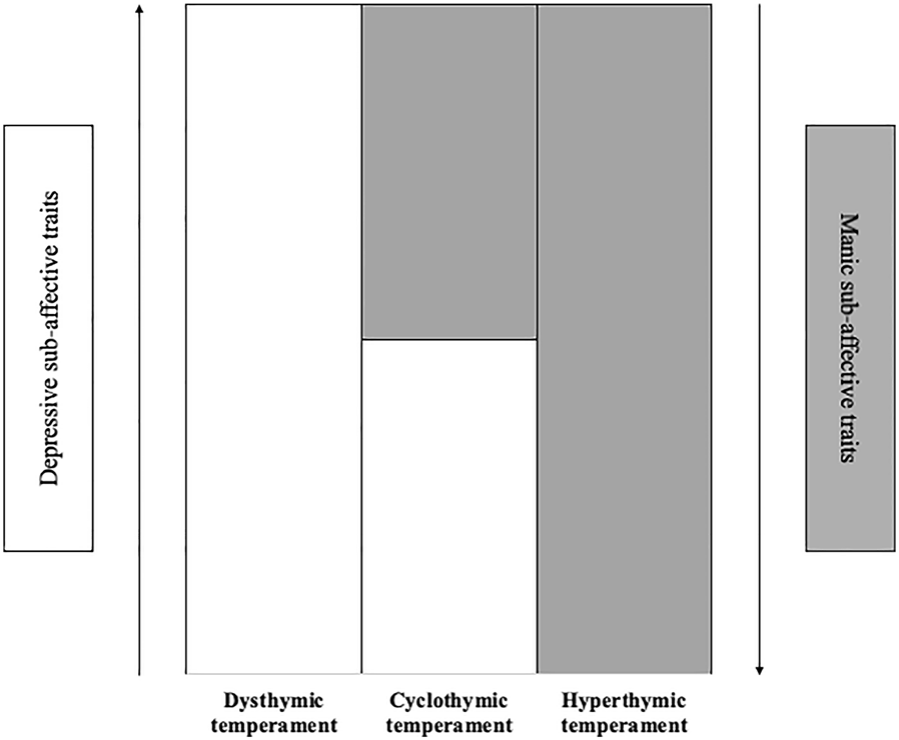
Affective temperaments classification based on different predominance of depressive and manic sub-affective states. Affective temperaments include dysthymic, cyclothymic, and hyperthymic temperaments, following the Clinical Research Diagnostic Criteria for Bipolar Illness (Ghaemi et al. 2022)

Moreover, a spectrum-based model may beneficially account for the placement of affective temperaments (Akiskal 1994; Akiskal et al. 1992), thus integrating clinical evidence with pioneering evolutionary theories in this field (Akiskal and Akiskal 2005). Since Emil Kraepelin (Kraepelin 1921) these were described as ‘fundamental states’ (e.g., constitutional moodiness and excitement), accompanying the free intervals of morbid phenomena related to the *manic-depressive insanity*. Temperaments are generally defined as early predispositions towards different emotions and ways of reacting to the environment, not amenable to change and more stable than personality (Rettew and McKee 2005). More specifically, affective temperaments should be considered subsyndromal, trait-related, predispositions towards specific affective states (Gonda and Vázquez 2014; Rihmer et al. 2010). What matters most for our understanding is that temperaments also seem to be contextualizable to some extent within a predominant polarity framework, though on sub-affective traits. Indeed, predispositions towards self-confidence and optimism or dysphoria and pessimism, define hyperthymic and dysthymic temperaments, respectively, while cyclic alternation and rapid shift between these sub-affective traits describe a cyclothymic temperament (Akiskal et al. 2005; Karam et al. 2023). Consistently, cyclothymic, hyperthymic, and dysthymic temperaments have been included among the Clinical Research Diagnostic Criteria for Bipolar Illness (Ghaemi et al. 2022) (Fig. 2).

Being able to shape emotional reactions, temperaments may influence the vulnerability to affective disorders, their related unipolar or bipolar course, as well as their specific predominant polarity (Gonda and Vázquez 2014; Fountoulakis et al. 2016; Simonetti et al. 2023). It is likely that people with a hyperthymic temperament may be more prone to MP or unipolar mania, while, in individuals with dysthymic temperament, DP or unipolar depression more often occur.

In this scenario, mixed features, as defined by DSM-5 (Bartoli et al. 2020; Na et al. 2021) or based on other conceptualizations (Malhi et al. 2016, 2017), or may emerge from manic symptoms interfering with a dysthymic temperament or, conversely, from depressive symptoms intruding into a hyperthymic temperament (Akiskal et al. 1998; Perugi et al. 1997). Similarly, other common specifiers of depressive episodes, such as anxious distress (Bartoli et al. 2024), may be the result of the interaction between temperament and mood state (Serafini et al. 2018).

Preliminary empirical data exploring the relationship between predominant polarity and affective temperaments are available. Since the late 1990s Henry et al. (1999) showed that the proportion of manic episodes positively correlated with hyperthymic temperament scores (B = 0.01; p = 0.04) and negatively correlated with dysthymic temperament scores (B =  − 0.02; p < 0.001), also after adjusting for age, sex, and education (B =  − 0.01; p = 0.002). In addition, Perugi et al., (2007) reported that people with unipolar mania might be more likely to have a hyperthymic temperament (74% vs. 57%) and less likely to have a dysthymic temperament (0% vs. 21%) than people with bipolar mania. However, Mazzarini et al. (2009) showed that people with MP and DP may have similar temperaments (either hyperthymic or cyclothymic), while people with unipolar depression have higher dysthymic temperament scores (p < 0.001) and lower hyperthymic and cyclothymic temperament scores (p < 0.001). More recently, Azorin et al. (2015), testing 228 patients who met criteria for a predominant polarity, found that both hyperthymic (OR 1.10; 95% CI 1.02–1.20) and cyclothymic (OR: 3.57; 95% CI 1.14–11.12) temperament scores were significantly associated with MP. However, no correlation between dysthymic temperament and DP was estimated (p = 0.74). In sum, studies investigating this relationship have shown a mixed interplay between predominant polarity and affective temperament, whose role as a determinant of the individual predisposition to depressive and manic features remains unclear.

## Epidemiological evidence on predominant polarity

Systematic reviews provide epidemiological data on predominant polarity (Carvalho et al. 2014; García-Jiménez et al. 2019; Bartoli et al. 2024). Carvalho et al. (2014) reported that a predominant polarity might be identified in approximately half of BD patients, with varying prevalence rates across studies, based on different eligibility criteria (namely the inclusion or the exclusion of people with BD-II). More recently, a meta-analysis (Bartoli et al. 2024) based on 28 studies, estimated similar rates of MP (30.0%) and DP (28.5%) in BD. Nonetheless, these estimates may be significantly influenced by medications, obviously shaping the longitudinal course of BD, the occurrence of mood relapses, and the related type of predominant polarity (Ilzarbe and Vieta 2023). In terms of clinical correlates, younger age, male gender, BD-I, psychotic features, earlier and manic onset were associated with MP, while depressive onset, number of mood episodes, and history of suicide attempts were associated with DP (Bartoli et al. 2024). Similar findings were proposed in another review (García-Jiménez et al. 2019), additionally showing that people with MP have a better response to atypical antipsychotics and mood stabilizers, whereas individuals with DP more often report comorbid anxiety disorders, mixed features, melancholic symptoms, and are treated with quetiapine or lamotrigine.

**Fig. 3:**
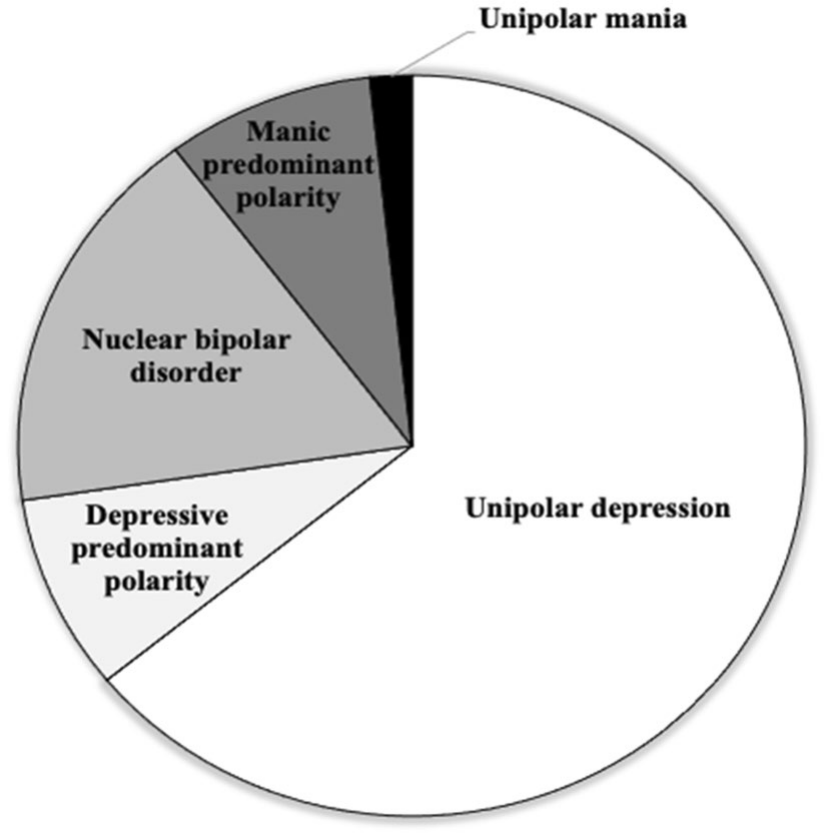
Estimated epidemiological distribution of different phenotypes of the affective spectrum. Estimates derived from available epidemiological data on prevalence rates of different affective phenotypes in general population: unipolar depression ≈ 4.5%; depressive predominant polarity ≈ 0.6%; nuclear bipolar disorder ≈ 1.2%; manic predominant polarity ≈ 0.6%; unipolar mania ≈ 0.1%

Information on the prevalence of predominant polarity in the context of the affective spectrum can be derived from available epidemiological studies on different affective disorders. According to the available data, a conservative estimate of 4–5% of the general population is likely to be affected by unipolar depression (Ferrari et al. 2013), while the lifetime prevalence of BDs is about 2–3% (Clemente et al. 2015). However, considering that predominant polarity would involve about half of subjects with BD and that MP and DP have comparable frequencies (Carvalho et al. 2014), it can be estimated that BDs with MP and DP would affect around 0.5–0.7% of the general population, respectively, while the nuclear subtype would occur in 1–1.5%. In addition, since more than 90% of patients with manic episodes will also develop depressive episodes (Baek et al. 2014), the prevalence of unipolar mania in general population is likely to be around 0.1%. From these figures, it can be argued that unipolar depression represents by far the most frequent phenotype of the affective spectrum, followed by nuclear BD, while unipolar mania is the least frequent form (Fig. 3).

There may also be some geographical differences in the frequency of predominant polarity in BD (Bartoli et al. 2024). In the absence of a comprehensive, rigorous epidemiological study, it is likely that the predisposition to depression or mania is influenced by genetic, cultural, and environmental factors. According to the ‘*Barcelona proposal*’ (Colom et al. 2006), it seems that in European studies, DP may be more common than MP, representing about 35–50% and 9–14%, respectively, of people with BD (Azorin et al. 2015; Albert et al. 2021; Volkert et al. 2014). Similarly, in a post-hoc analysis on 788 patients randomized to an 8-week trial of treatment with olanzapine, olanzapine/fluoxetine, or placebo, a predominant polarity was found in almost half of patients (Vieta et al. 2009). Among them, a 2.7-fold excess of DP over MP (34% vs. 12%) was found, regardless of sex and treatments (Vieta et al. 2009). On the other hand, rates of predominant polarity seem to follow an opposite trend in non-Western countries (Bartoli et al. 2024). A multicenter study from India, including 773 participants with at least 10 years of illness, estimated that around 20% of the patients with BD had DP, while almost 50% of them had MP (Grover et al. 2021). In addition, data on patients consecutively admitted to the National Institute of Mental Health and Neurosciences of Bangalore (India), showed that 79% of study participants with BD-I had MP and only 13% had DP (Rangappa et al. 2016). Similarly, a retrospective investigation on subjects with either BD-I or BD-II admitted to different university hospitals in Republic of Korea, showed higher rates of MP than DP in patients treated with lithium (38% vs. 10%), valproate (39% vs. 17%), and lamotrigine (22% vs. 18%) (Woo et al. 2020). These data seem to support the hypothesis that in non-Western countries unipolar mania and manic episodes in BD occur more frequently (Angst and Grobler 2015), though an alternative explanation may be that depression is less frequently diagnosed perhaps because its signs are less evident and are more likely to be subsumed within cultural expressions of distress (Malhi and Byrow 2018).

## Treatment implications of predominant polarity

**Fig. 4:**
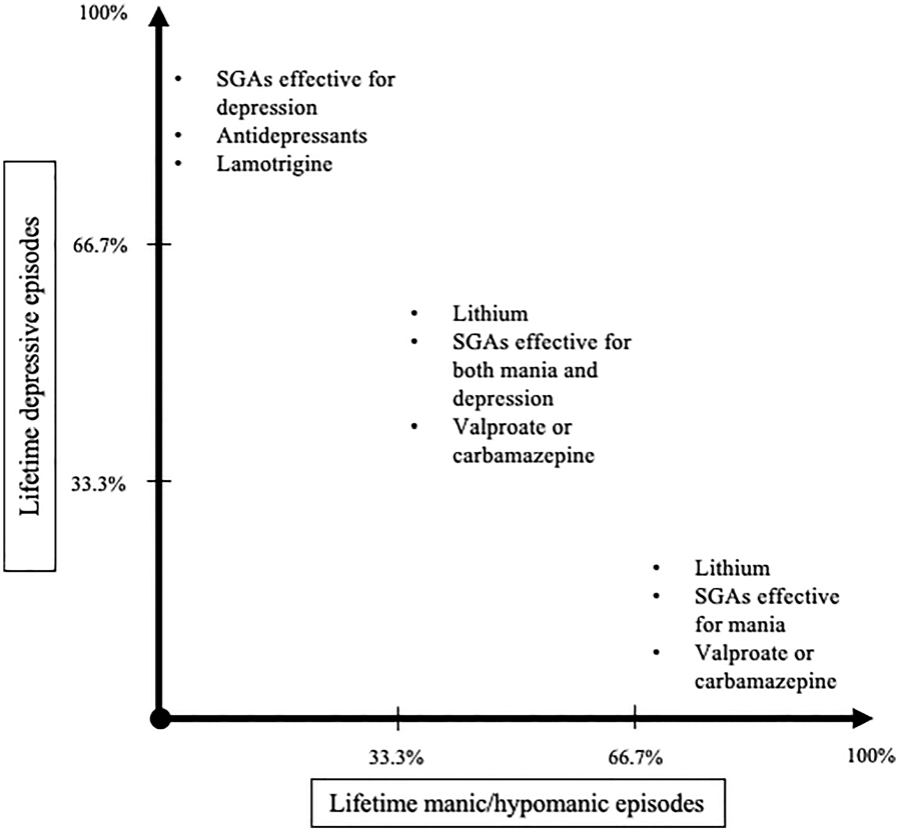
Pharmacological treatments by predominance of depressive and manic episodes. SGAs: second-generation antipsychotics

Clarifying the predominant polarity of the single patient may provide useful information to clinicians for the early identification of possible trajectories of BD and guide them in their selection of interventions for maintenance treatment. Indeed, different predominant polarities may significantly influence the individual response to both acute and prophylactic treatments of BD (Carvalho et al. 2014; Bartoli et al. 2018; Carvalho et al. 2014; Goes 2023). Nonetheless, a spectrum-based classification accounting for predominant polarity in place of conventional subtypes of BD (BD-I and BD-II), should not be considered a ground-breaking shift, rather the clarification of the approaches implicitly followed by clinicians in their practice. For instance, the absence of any history of manic/hypomanic episodes in people with depression (*unipolar depression*) generally calls for the selection of an antidepressant mono- or multi-therapy (Henssler et al. 2022); the combinations of antidepressants with agents, such as, e.g., quetiapine or lamotrigine (Hashimoto et al. 2021; Kadakia et al. 2021), are often used in people with BD and frequent depressive episodes (*DP*); the mood stabilizing effects of lithium and/or valproate (Geddes et al. 2010; Fountoulakis et al. 2022; Kang et al. 2020) are typically advocated for people with a balanced cyclicity (*nuclear BD*); a combination of mood stabilizers with antipsychotic agents with antimanic properties (such as risperidone or aripiprazole) (Kishi et al. 2022), even in their long-acting formulation (Bartoli et al. 2023b; Mauri et al. 2020), is likely to be prescribed for people with a manic propensity (*MP* and *unipolar mania*) (Fig. 4).

Predominant polarity may also tailor clinical choices involved in the often debated and controversial use of antidepressant agents for bipolar depression (Gitlin 2018). Specifying the predominant polarity would make clearer that (*i*) the use of antidepressants associated with mood stabilizers may be recommended in patients with DP, whereas (*ii*) caution is required in people with nuclear BD, and (*iii*) antidepressants might be used for a limited time, or not at all, in people with acute depression across a lifetime MP.

In keeping with the need to translate the predominant polarity concept into clinical practice, it has been proposed to implement the concept of ‘*Polarity Index*’ of different drugs (Popovic et al. 2012). This defines the ratio between antimanic versus antidepressant properties of single pharmacological agents used for the maintenance treatment of BD (Popovic et al. 2012, 2014). Values > 1.0 indicate greater antimanic prophylactic efficacy, whereas values < 1.0 greater antidepressant efficacy. According to a recent systematic review and meta-analysis of randomized controlled trials (Nestsiarovich et al. 2022), the drugs approved for BD are generally more effective in preventing mania than depression (overall Polarity Index: 1.38). In particular, Polarity Index values were 2.29 for lithium, 1.57 for second-generation antipsychotics, and 0.38 for anticonvulsants. Nonetheless, despite its relatively easy adaptation into clinical practice, the practical use of the Polarity Index has been questioned given that it has not been possible to estimate specific values for several psychopharmacological agents (Popovic et al. 2012) and issues about its validity and reliability have been raised (Alphs et al. 2013). Interestingly, the Polarity Index was also estimated for non-pharmacological interventions for BD (Popovic et al. 2013), with values less than one (< 1) for cognitive-behavioral therapy, family-focused therapy, and psychoeducation, while caregiver group psychoeducation and brief technique-driven interventions showed a Polarity Index of greater than one (> 1).

## Conclusions

About half of people with BD show a predominant polarity during their illness, with similar rates for MP and DP. Although various factors are likely to influence its course, affective temperaments, including dysthymic, cyclothymic, and hyperthymic ones, may play a key role in determining an individual’s propensity. The conceptualization of predominant polarity within the affective spectrum, accounting for either unipolar (*ultra-predominant* polarity) or bipolar course (nuclear, MP, and DP subtypes) disorders, may reconcile previous theoretical views on BD (Angst 2007; Colom et al. 2015; Akiskal 1996; Ghaemi et al. 2022). In addition, while the utility of the Polarity Index in clinical practice is yet to be established, it is likely that accounting for predominant polarity, instead of conventional approaches such as the differentiation of BD-I and BD-II, may be useful when seeking to clarify the natural history of the disorder and inform the selection of suitable interventions. However, ultimately, as yet, additional research is needed to better characterize predominant polarity and clarify its role in particular, as a determining factor with respect to the course, outcome, and treatment response of BD.

## Data Availability

No datasets were generated or analyzed for this study.
